# Precision Therapeutics: A Wider Aspect of Personalized Medicines

**DOI:** 10.7759/cureus.90787

**Published:** 2025-08-23

**Authors:** Anju Kaushal

**Affiliations:** 1 Science/Microbiology, Panjab University, Chandigarh, IND; 2 Science/Biotechnology, New Zealand Organization for Quality, University of Auckland, Auckland, NZL

**Keywords:** data set, infection, nephrocheck testing, precision medicine, somatic mutations brca 1 & 2

## Abstract

"Precision medicine" will become an intrinsic part of the healthcare system in the near future, and some elaborated features are discussed in this editorial. In recent years, the rise in therapeutic disparities has created significant challenges for "one size fits all" medicines. Modern science has greater potential to deliver more personalized care in terms of medication prescription, patient treatment, health outcomes, and data storage and analysis, while remote communications are becoming regular practice in medical science. Pharmacogenetics and pharmacogenomics have emerged, reshaping precision diagnosis with broad capabilities to depict the whole biocycle in individuals. However, defining gene expression standards remains an area requiring further improvement. Additionally, understanding biological processes, improving diagnostic accuracy, considering ambient environments, optimizing therapies, ensuring standard patient care, enhancing data management, and fostering collaborations among clinicians, researchers, staff, and patients will play key roles in this new era of medicine. Personalized management of gene disorders, cancer, and infectious diseases could help avoid undue drug interactions and adverse events, thereby saving lives. Small-group trials are being conducted based on genetic traits and lifestyle factors that specify certain geographies, while large-population trials are being contextualized with outbreaks of similar pathogenic clades.

## Editorial

The advancements in pharmacogenetics and pharmacogenomics have expanded from precise disease diagnosis, disease risk predictions, targeted treatments, and evaluating disease prognosis to pathogen surveillance, with the potential to understand the impacts of outbreaks, enabling us to make real-time decisions. The capabilities of companion testing in imaging, drug delivery, and monitoring the drug response have emerged to eliminate the occurrence of adverse events during treatment. Sequencing and molecular docking OMICS-based testing may hold the promise of predicting health risks. Building models of personalized ecosystems representing precision diagnosis and therapy will push the boundaries to establish robust systems of data repositories, IT infrastructure, IoTs, including AI and machine learning, for faster communication to advance predictive analysis and make real-time decisions for appropriate patient care, which would certainly lessen the economic burden on the healthcare sector. Collaborations among specialists, clinicians, general practitioners, researchers, scientists, staff members, and patients improve the ability to understand patients’ needs, analyze datasets, and develop targeted therapies, which could be an approach for implementing robust strategies. Healthcare studies based on individuals, small groups, or large populations, while considering genetic and molecular therapies, are entirely dependent on the specific disease risk and treatment therapy, ranging from genetic disorders and cancers to autoimmune and infectious diseases [1]. This collection aims to present a holistic view of precision medicine and its applications through various clinical case studies, focusing on controlling disease severity, improving diagnostic accuracy, optimizing doses, minimizing drug cross reactions to halt neurological symptoms, conducting pre-surgical molecular checks for better patient care, and analyzing large datasets.

Khalpey et al. (2024) explained that acute kidney injuries occur in 22-30% of patients undergoing coronary bypass surgery. The authors developed two novel nephro-check biomarkers, growth factor binding protein 7 (IGF BP-7) and tissue inhibition of metalloproteinase 2 (TIMP2), which are considered better predictors to detect early kidney stress. Hence, early interventional personalized strategies help guide body fluid management during surgery. However, under conventional practices, these nephron checks are not required comparatively [2]. In the case of prostate cancer, BRCA1 and BRCA2 somatic mutations with HRR have been managed with poly ADP-ribose inhibitors, as recommended by NCCN and ESMO (cancer management societies). Interestingly, in a phase III PROPEL trial, the drug olaparib plus abiraterone was found to benefit patients harboring BRCA1/2 with and without HRR deficiency, studied in metastatic castration-resistant prostate cancer. The cohort of 42 patients in Portugal revealed approximately 3.1% uncertainty according to universal testing, which would certainly demand more studies to eliminate target drug inefficiencies [3].

Extensive dataset analysis through repositories could help guide the optimal use of acute medications in large populations. The REZULT database used by Japan System Technologies Co. Ltd., Tokyo, Japan, conducted two investigational studies based on prescription profiles to treat migraine-related illness utilizing prophylactic medicines such as lomerizine, propranolol, valproic acid, amitriptyline, and verapamil, along with non-steroidal anti-inflammatory drugs (NSAIDs) and others. However, all the patients belonged to the working class, a direct indication that socioeconomic factors influenced the overuse of these acute medications [4].

It is essential to determine whether cross reactions of two or more drugs interfering with each other’s metabolism can exacerbate neurologic disorders. A brucellosis patient treated with rifampicin was found to have increased metabolism of valproic acid, consequently experiencing several seizure attacks. The discontinuation of rifampicin with subsequent adjustment using carbamazepine and lamotrigine, at dose levels of 0.3 g and 75 mg BID, respectively, eventually helped in controlling the neurological attacks [5].
